# Characteristics, sources and potential ecological risk of atmospheric microplastics in Lhasa city

**DOI:** 10.1007/s10653-024-02125-w

**Published:** 2024-07-29

**Authors:** Zimeng Guo, Junyu Chen, Hanyue Yu, Qiangying Zhang, Bu Duo, Xiaomei Cui

**Affiliations:** Laboratory of Biodiversity and Environment on the Qinghai-Tibetan Plateau, Ministry of Education, School of Ecology and Environment, Tibet University, Lhasa, 850000 China

**Keywords:** Lhasa, Atmospheric environment, Microplastics, Occurrence features, Laser infrared

## Abstract

Atmospheric microplastics are important contributors to environmental contamination in aquatic and terrestrial systems and pose potential ecological risks. However, studies on atmospheric microplastics are still limited in urban regions of the Tibetan Plateau, a sentinel region for climate and environmental change under a warming climate. In this study, the occurrence and potential ecological risk of atmospheric microplastics were investigated in samples of suspended atmospheric microplastics collected in Lhasa city during the Tibetan New Year in February 2023. The results show that the average abundance of atmospheric microplastics in Lhasa was 7.15 ± 2.46 MPs m^−3^. The sizes of the detected microplastics ranged from 20.34 to 297.18 μm, approximately 87% of which were smaller than 100 μm. Fragmented microplastics (95.76%) were the dominant shape, followed by fibres (3.75%) and pellets (0.49%). The primary polymer chemical components identified were polyamide (68.73%) and polystyrene (16.61%). The analysis of meteorological data and the backwards trajectory model indicated the air mass in Lhasa mainly controlled by westwards, and the atmospheric microplastics mainly originated from long-distance atmospheric transport. The potential ecological risk index assessment revealed that the atmospheric microplastic pollution in Lhasa was relatively low. This study provides valuable insights and a scientific foundation for future research on the prevention and control of atmospheric microplastic pollution in Lhasa and other ecologically sensitive cities.

## Introduction

Since the mid-twentieth century, the global production of plastics has increased significantly, driven by their light weight, affordability, durability, and water resistance. An increase in production, coupled with inadequate recycling, degradation, and reuse methods, has led to an increase in the accumulation of plastics in the environment (Rodrigues et al., 2018).

Statistics reveal that approximately 79% of plastic waste accumulates in landfills or the natural environment (Geyer et al., 2017; Thacharodi et al., 2024). Under the influence of physical, chemical, and biological factors, larger plastic pieces gradually break down into microplastics, which are typically defined as plastic particles with size smaller than 5 mm (Thompson et al., 2004). Microplastics are categorized into primary and secondary microplastics. Primary microplastics are industrially produced particles such as microbeads in cosmetics, while secondary microplastics are derived from the degradation of larger plastics (Hidalgo-Ruz et al., 2012; Bhat et al., 2024d). Due to their small size, low density, and light weight, microplastics can be easily airborne, resulting in their widespread distribution and accumulation in various environmental matrices, including water, land and air (Bhat et al., 2023). Once airborne, microplastics can travel long distances, reaching remote and even polar regions (Allen et al., 2019; Bergmann et al., 2019; Zhang et al., 2019). Furthermore, the extensive surface area of microplastics makes them potential carriers of toxic substances (e.g., persistent organic compounds, heavy metals and antibiotics), posing significant risks to ecological systems and human health through bioaccumulation (Wang et al., 2020; Bhat et al., 2024a).

Recognizing the gravity of the issue, the United Nations Environment Programme (UNEP) classified microplastic pollution as one of the top 10 emerging environmental challenges globally in 2014 (United Nations Environment Programme, 2014). Great efforts have been made to investigate microplastic pollution in aquatic (Besseling et al., 2017; Fu et al., 2019; Gillibert et al., 2019; Li et al., 2018; Suaria et al., 2020; Zhang et al., 2018; Bhat et al., 2024c) and terrestrial environments (Du et al., 2020; He et al., 2018; Ren et al., 2021). However, atmospheric microplastic pollution has received less attention. In 2015, research on atmospheric microplastics was launched in Paris, France (Dris et al., 2015), a relatively late start. In recent years, although research on microplastics has increased significantly, it has focused mainly on occurrence characteristics, sources, transport and sedimentation, etc., and research on atmospheric microplastics is still very limited and unevenly distributed, which are mainly concentrated in a few regions. Moreover, there are few studies on remote areas, especially special areas at high altitudes and with ecological sensitivities, which limits the understanding of atmospheric microplastic migration and ecological risks.

Known as the Third Pole and the Asian Water tower, Tibet's unique geographical position significantly influences atmospheric circulation and the hydrological cycle both in Asia and even globally. Its sparse population and correspondingly low levels of atmospheric pollutants offer a distinct advantage for accurately pinpointing the origins of microplastics. Thus, it is crucial to investigate the sources, pathways and transformation mechanisms of atmospheric microplastics in Tibet. Lhasa, Tibet's capital (29.65° N, 91.13° E), situated north of the Himalayas, is increasingly affected by human activities, particularly tourism. To comprehensively understand the occurrence characteristics of atmospheric microplastics in such isolated regions, we used laser direct infrared (Agilent 8700 LDIR) spectroscopy for the first time to analyze the occurrence characteristics of suspended atmospheric microplastics in Lhasa and their potential sources. Furthermore, we carried out a potential ecological risk assessment. This study aimed to provide essential data for a deeper exploration of the characteristics and risk assessment of suspended atmospheric microplastics under the unique alpine and hypoxic conditions of Tibet.

## Materials and methods

### Research area and sampling methods

**Fig. 1:**
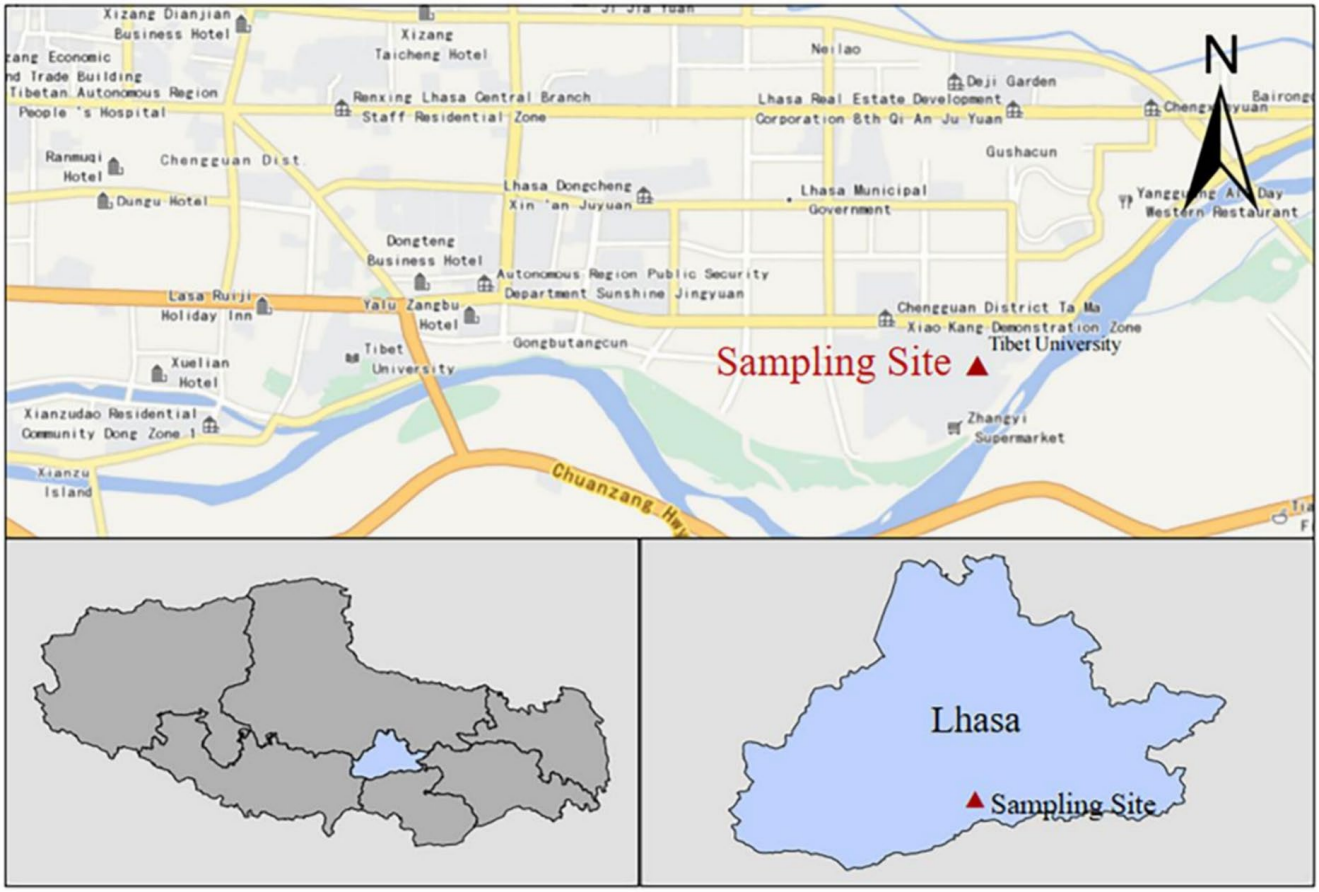
Map showing the locations of the sampling sites

As an economic and cultural center of the Tibet Autonomous Region, Lhasa is located in the south-central part of the Qinghai—Tibet Plateau, with a permanent population of 867,900 and a gross domestic product (GDP) of 67.816 billion yuan in 2020. The sampling site is located on the roof of a four-floor experimental building on the Najin Campus of Tibet University in Lhasa (29°38′44.34″N, 91°10′52.81″E), approximately 12 m above the ground and 3662 m above sea level (3650 m above ground level in Lhasa), with no surrounding tall buildings and far from industrial pollution sources (Fig. 1).

The sampling time was from February 20th to February 22nd, 2023. Samples collected during the three days were recorded as LS01, LS02 and LS03. February 21st was the Tibetan New Year and folk activities such as religious activities and fireworks displays impacted the atmospheric environment. The samples were collected using an intelligent medium-flow total suspended particle sampler (1037B, Qingdao Laoying) for 24 h with an intake flow rate of 100 ± 0.1 L/min. To avoid randomness, a parallel sample is also collected. Before sampling, a Whatman GF/A membrane with an aperture size of 1.6 μm and a diameter of 90 mm was carefully placed in an aluminium alloy divider at the upper part of the instrument, then the sampler was placed horizontally on an aluminium alloy tripod at a height above the ground of approximately 1.7 m (the height of universal human breathing). For delayed sampling, the sampler was quickly moved downwind to avoid clothing contamination after startup. Once sampling was completed, stainless steel tweezers were used to quickly transfer sample membranes to aluminium foil and wrap them.

The altitude, latitude, longitude, sampling time, sampling volume and meteorological conditions of the sampling site such as temperature, humidity, wind speed, wind direction and air pressure were recorded.

### Sample pretreatment

Both sides of the sample membrane were rinsed with 1.7–1.8 kg/L ZnCl_2_ (premium pure) solution. The particles on the sample membrane were flushed into a 100 mL beaker, stirred thoroughly for 2 min, then incubated for 12 h. The supernatant was then transferred to another beaker and 60 mL of 30% H_2_O_2_ solution was added to remove the organic matter. The mixture was stirred thoroughly and left to stand for 24 h to allow the H_2_O_2_ to fully react with the organic matter. The solution treated with H_2_O_2_ was vacuum filtered with a steel mould, and the obtained filter membrane was immersed in an ethanol solution for ultrasonic treatment, so that the substances on the filter membrane were dispersed in the ethanol solution. The filter membrane was removed and washed multiple times with ethanol solution, the ethanol solution was concentrated to approximately 100 μL, then added dropwise onto high reflection glass. LDIR testing was conducted after the ethanol had completely evaporated.

### Sample identification

LDIR was used for the measurement of microplastics. A microplastic spectrum library established in particle analysis mode was selected by setting up automatic testing methods, and the measured chemical spectrum was compared with the reference spectra for polymers. Those with a matching value greater than 65% were confirmed as microplastics, while nonplastic samples were excluded (Wesam, et al., 2022).

### Backwards trajectory

The backwards trajectory is the actual path of an air mass moving in the atmosphere and usually refers to the path of air mass in a specific period of time before reaching a certain point. The 72 h backwards trajectory of the airflow arriving at the study area during the sampling period was simulated, the meteorological data were obtained from the GDAS, and the simulated height was set to 500 m.

### Ecological risk assessment

The potential ecological risk index (PERI) method (Everaert, et al., 2018) was used to assess the risk posed by atmospheric microplastics in Lhasa. The potential ecological risk index method was initially proposed by the Swedish scientist Hakanson and has been widely used in the study of heavy metal pollution. At present, relevant research has improved the potential ecological risk index method for heavy metals, forming a potential ecological risk index method for microplastic ecological risk research (Xu et al., 2018). The calculation formula is as follows:

1 $${\text{Microplastic pollution index}}:C_{f}^{i} = \frac{{C^{i} }}{{C_{r}^{i} }}$$

2 $${\text{Ecological hazard factors of single microplasticpolymers}}:T_{r}^{i} = \frac{{P_{i} }}{{C_{i} }} \times S_{i}$$

3 $${\text{Potential ecological risk index of single microplastic polymer}}:E_{r}^{i} = T_{r}^{i} \times C_{f}^{i}$$

4 $${\text{Ecological risk index of various microplastic polymers}}:{\text{RI}} = \mathop \sum \limits_{i = 1}^{n} E_{r}^{i}$$

where, ${C}_{f}^{i}$ is the pollution index of polymer *i*; ${C}^{i}$ is the observed measured polymer concentration;${C}_{r}^{i}$ is the standard reference value of polymer *i*. In this experiment, the minimum abundance of all samples (4.31 pieces m^−3^) was selected as the reference value; ${S}_{i}$ is the harm index of microplastic polymers; ${T}_{r}^{i}$ is the ecological toxicity response factor of microplastic *i*; ${P}_{i}$ is the concentration of microplastic polymer* i*;${E}_{r}^{i}$ is the potential ecological risk index of microplastic *i*; RI is the ecological risk index of various microplastic polymers; and n is the number of types of microplastic polymers contained in the test sample.

### Quality assurance and statistical analysis

In order to minimize exogenous contamination, we have taken the following measures. Plastic materials were avoided as much as possible, Stainless steel tweezers are used during sample collection, disposable powder-free latex gloves are worn, and the sampling membrane is wrapped in aluminum foil to avoid contact between samples and the ground or contamination between samples. The experiment was carried out in a clean room to avoid the interference of microplastics in the air. During the experiment, to avoid background contamination, the experimenter wore experimental clothing to prevent fibre shedding. All laboratory instruments and equipment were rinsed with ultrapure water before use, and wrapped in aluminum foil immediately after use. The reagent solutions used were filtered through a 0.45 μm filter before use. Samples for observation and identification after concentration are kept in glass bottles and placed in clean storage cabinets. In the process of analysing the microplastic statistics and calculations, the same microplastic samples used for the blank samples were subtracted (Bhat et al., 2024e). The data analysed in the current study were processed using Microsoft Excel 2021 and Origin 2021. Principal component analysis was used to explore the relationships between the abundance of SAMPs and meteorological variables during the sampling period. Single factor analysis of variance was used for statistical processing, and *P* < 0.05 indicated a significant difference.

## Results and discussion

### Abundance of SAMPs

**Fig. 2:**
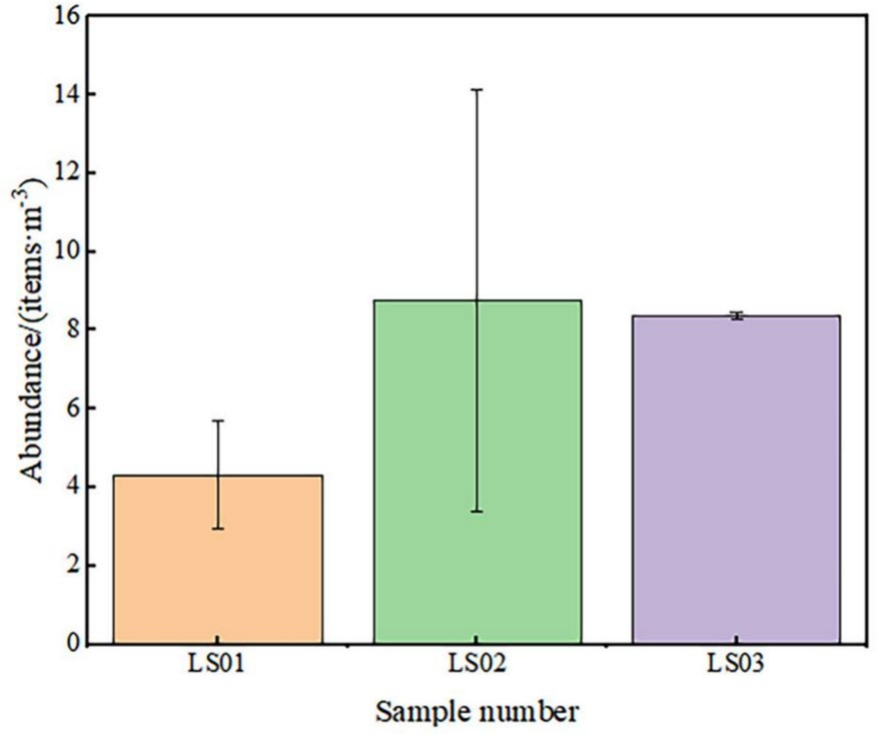
Abundance values of SAMPs in Lhasa city

In this study, 3080 microplastic particles were detected over all samples, indicating that SAMPs are widespread in the Lhasa atmosphere. The abundance ranged from 4.32 to 8.77 MPs m^−3^, with an average abundance of 7.15 ± 2.46 MPs m^−3^ and a median of 8.36 MPs m^−3^ (Fig. 2). Compared with the median of 0.9 MPs m^−3^ found in air samples from Paris (Dris, et al., 2017), the pollution level in this study was relatively high. This may be because automatic identification using LDIR more easily captures small microplastics than does the use of FTIR methods. On the other hand, the dry winter in Lhasa, which has less precipitation, made microplastics settling on the ground prone to resuspension and entering the air, leading to a high abundance of SAMPs. Among the samples, the abundances found in samples LS02 (February 21, 2023) and LS03 (February 22, 2023) were similar and that of LS02 was slightly greater, both approximately twice that of LS01 (February 20, 2023), which may be due to the impact of biomass and fireworks on the atmospheric environment on February 21, the Tibetan New Year.

### Size characteristics of SAMPs

**Fig. 3:**
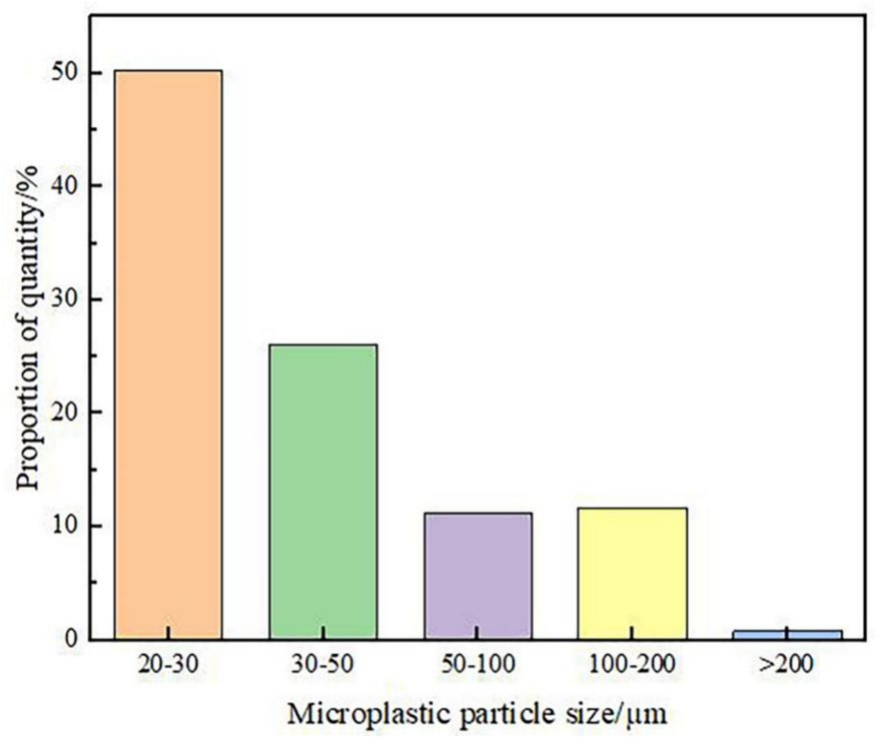
Particle size distribution of the SAMPs

This study tested microplastic particles with sizes between 20 and 500 μm. The results show that the particle size of microplastics in the atmospheric environment of Lhasa was generally small, with a maximum particle size of 297.18 μm, a minimum particle size of 20.34 μm, and an average particle size of 49.09 μm. According to the five gradients of 20–30 μm, 30–50 μm, 50–100 μm, 100–200 μm and > 200 μm, it can be seen from Fig. 3 that the SAMPs with 20–30 μm and 30–50 μm particle sizes accounted for a relatively large proportion, 50.24% and 26.02%, respectively, those > 200 μm accounted for the least, and the proportion of microplastics with particle sizes of 50–100 μm and 100–200 μm was not much different. In addition, the overall particle size of PS was relatively large, mainly in the range of > 50 μm, approximately 99% and 64.36% in the range of 100–200 μm, and was not detected in the range of 20–30 μm. The four types of microplastics, polyethylene (PE), polypropylene (PP), polyurethane (PU) and acrylates (ACR), were detected only in the < 100 μm particle size range. Among all microplastic types, PA had the widest particle size distribution, followed by polyvinyl chloride (PVC) and polyethylene terephthalate (PET). Overall, the number of SAMPs decreases with increasing particle size, which is the same as the conclusion of most studies. The particle size of microplastics is an important influencing factor for their migration and fate in environmental media (Besseling, et al., 2017). Compared with the particle size of microplastics in water and sediments, the particle size of microplastics in the atmospheric environment is significantly smaller (Zhang, et al., 2020), possibly because microplastics with smaller particle sizes are more likely to float in the atmospheric environment.

### Shape characteristics of SAMPs

The existing shapes of microplastics mainly include pellets, fragments, fibres, films and foam (Fahrenfeld, et al., 2018; yuan, et al., 2019). LDIR can only make rough judgements on the shape of microplastics based on two indicators (roundness and hardness) and can preliminarily distinguish among three shapes (pellets, fibres, and fragments) (Adrián, et al., 2022).

**Fig. 4:**
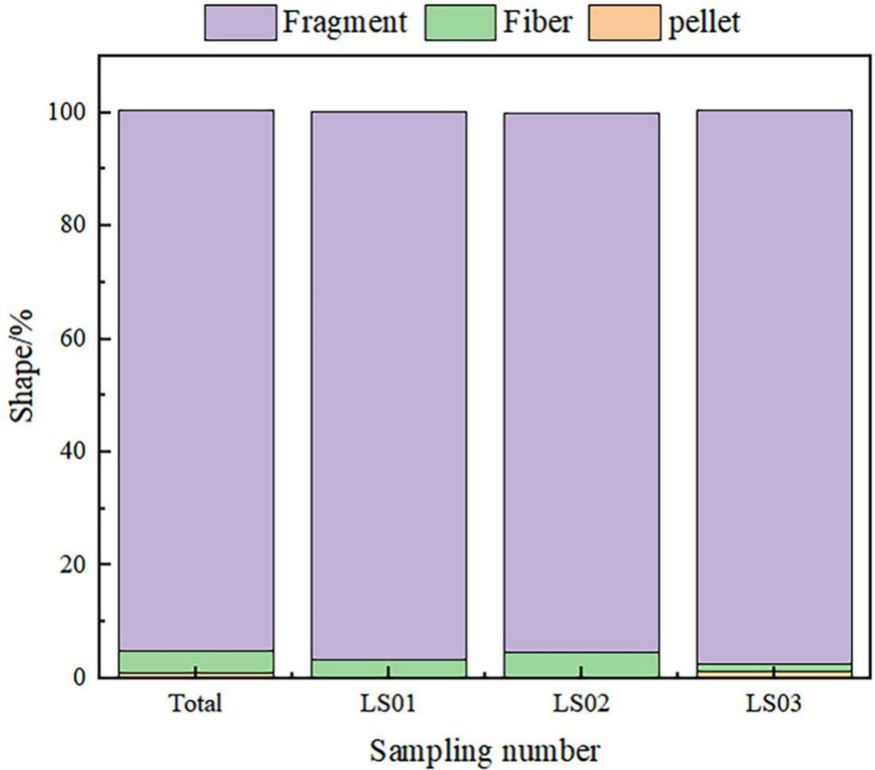
Shape distribution of SAMPs

The results show that the shape of microplastics in the suspended atmosphere in Lhasa was dominated by fragments, followed by fibres and pellets, accounting for 95.76%, 3.75% and 0.49%, respectively (Fig. 4). The morphological characteristics of the microplastics in each sample were basically the same as the overall shape characteristics, but there was a slight difference: there was no pellet morphology in samples LS01 and LS02.

Most studies have shown that fibres are the main shape of microplastics in regions such as Dongwan, China (Cai, et al., 2017), Yantai, China (Zhou, et al., 2017), London, the UK (DRIS, et al., 2017), and Paris, France (Wright, et al., 2020). However, the results of studies in Hamburg, Germany (Klein, et al., 2019), the Niulibis Mountains in France (Allen, et al., 2019), and Beijing, China (Liu, et al., 2022), showed that fragments are dominant. This difference may be due to the differences in microplastic products in different regions. Fibre microplastics typically originate from the textile industry and household textile washing, while fragmented microplastics are caused by the degradation of primary plastic products (Bhat, et al., 2024b , c; Xu, et al., 2022). In addition, climate conditions have an impact on the generation and spread of microplastics. In general, fragmented microplastics are produced by the decomposition of larger particles by mechanical abrasion and UV exposure (Bonifazi, et al., 2023; Wang, et al., 2022), so the atmosphere of Lhasa, which has a long period of sunshine, is mostly composed of fragmented microplastics.

### Composition analysis of the SAMPs

LDIR was used to identify the polymer composition of the SAMPs samples, involving a total of 22 different polymer types. The results indicate that PA was the dominant polymer, accounting for 68.73%, followed by PS, which together accounted for more than 85%. Eleven types of microplastics were detected in both LS01 and LS03, while a total of 16 types were detected in LS02. There were significant differences in the types of microplastics and polymers collected at different times (Table 1). There were five common polymer types in the three samples, namely, PA, PP, PS, PU and PVC, among which the detection rates of PA and PS were relatively high. In addition to the upper five polymers, two types of polymers (polyimide and PE) were detected in LS01 and LS02, and four types of polymers (melamine cyanurate (MCA), PET, polyisoprene and ACR) were also detected in LS02 and LS03.

**Table 1:** Polymer composition of SMPs in Lhasa

Sample	Composition and proportion
LS01	PA: 62.29%, PP: 1.64%, PS: 25.41%, PU: 1.64%, PVC: 1.64%, Polyimide: 0.82%, PE: 3.28%, Vinyl Bistearamide (EBS): 0.82%, Polybutadiene: 0.82%, Ethylene acrylic acid (EAA): 0.82%, Styrene Isopentene Styrene (SIS), 0.82%
LS02	PA: 67.47%, PP: 0.40%, PS: 18.25%, PU: 1.19%, PVC: 0.40%, Polyimide: 0.40%, PE: 1.98%, MCA: 0.40%, PET: 1.19%, Polyisoprene: 1.98%, ACR: 3.17%, Ethylene dioleic amide (EBO): 0.79%, Polymethyl methacrylate (PMMA): 0.40%, Polycarbonate (PC): 1.19%, Polytetrafluoroethylene (PTFE): 0.40%, Polyvinyl Butyral (PVB): 0.40%
LS03	PA: 73.32%, PP: 2.08%, PS: 10.42%, PU: 1.67%, PVC: 0.42%, MCA: 0.83%, PET: 5.42%, Polyisoprene: 0.42%, ACR: 3.75%, Polycaprolactone: 1.25%, Polylactic acid (PLA): 0.42%

**Fig. 5:**
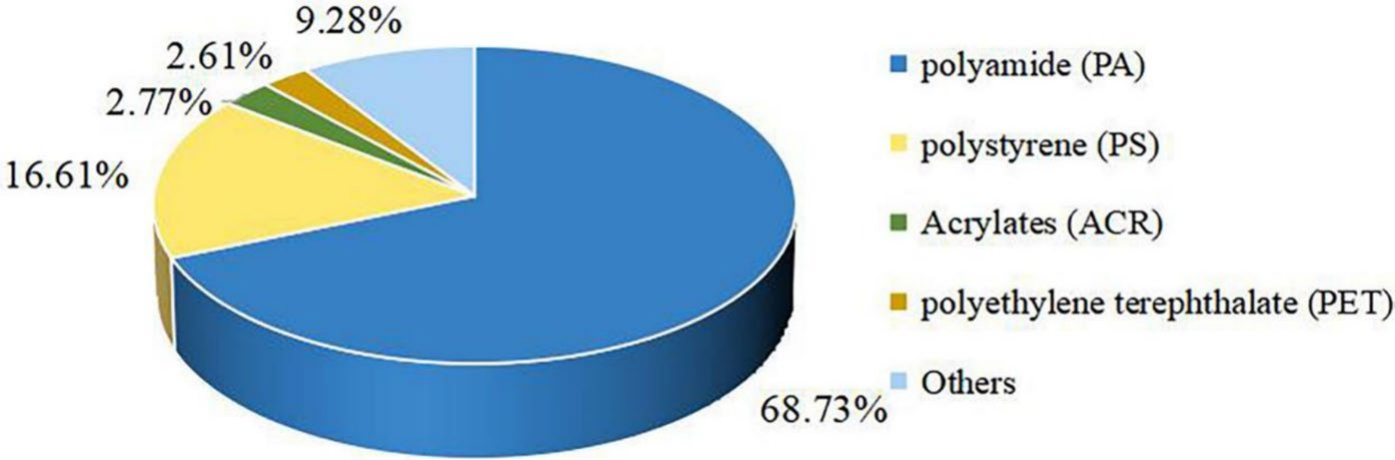
Distribution of Polymer Types in SAMPs

Overall, more than half of the microplastics detected in Lhasa were PA, followed by PS (16.61%). ACR and PET had similar quantities, accounting for 2.77% and 2.61%, respectively. Figure 5 shows the detection rates of different types of microplastics. Therefore, focusing on the potential sources of PA and PS plastics in the atmosphere of Lhasa may be highly important for further improving its air environment.

Different types of microplastics have different polymer compositions and their sources are also broad and diverse (Zhao, et al., 2019). Natural particulate matter may come from plant and agricultural emissions while synthetic microplastics may come from synthetic textiles and urban dust (Prata., 2018). PA, also known as nylon, mainly originate from the shedding of human clothing or agricultural tools. Previous studies have shown that washing clothes produces a large amount of microplastics (Browne, et al., 2010). In addition, drying textiles such as clothes can also cause microplastic pollution (Song, et al., 2017). PS is easy to process and mould, has good thermal insulation, and is often used in manufacturing industries such as the disposable plastic tableware and packaging industries (Di, et al., 2019). PET, also known as polyester, is a common plastic with excellent elasticity and wear resistance, and is widely used in the textile industry (Hidalgo-Ruz, et al., 2012). A 2022 study in the city of São Paulo (Amato-Lourenço, et al., 2022) and a study in Antarctica (Aves, et al., 2022) of the same year showed that the atmosphere contains the highest levels of PET.

### Potential sources of atmospheric microplastics

**Fig. 6:**
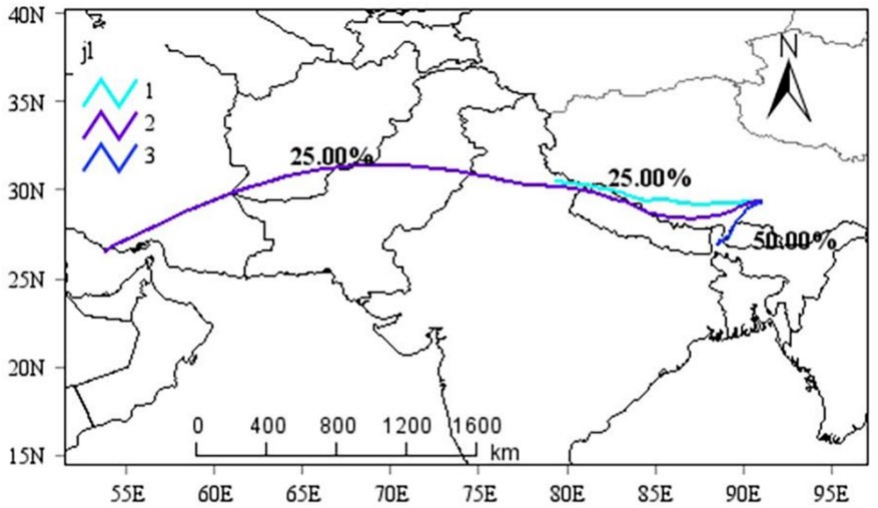
Simulated clustering analysis of atmospheric backwards trajectories during sampling periods

Through the simulation and cluster analysis of the atmospheric backwards trajectory in Lhasa, it is found that the air mass is obviously westerly, and the airflow trajectory is divided into three categories (Fig. 6).One originates from northeastern India and reaches Lhasa via Bhutan, one originates from northwestern India and reaches Lhasa via Nepal, and the other originates from distant southwest Asia and reaches Lhasa via Iran, Afghanistan, Nepal and other regions. This indicates that microplastics in the atmosphere of Lhasa are mainly transported through the atmosphere over long distances across regions.

**Fig. 7:**
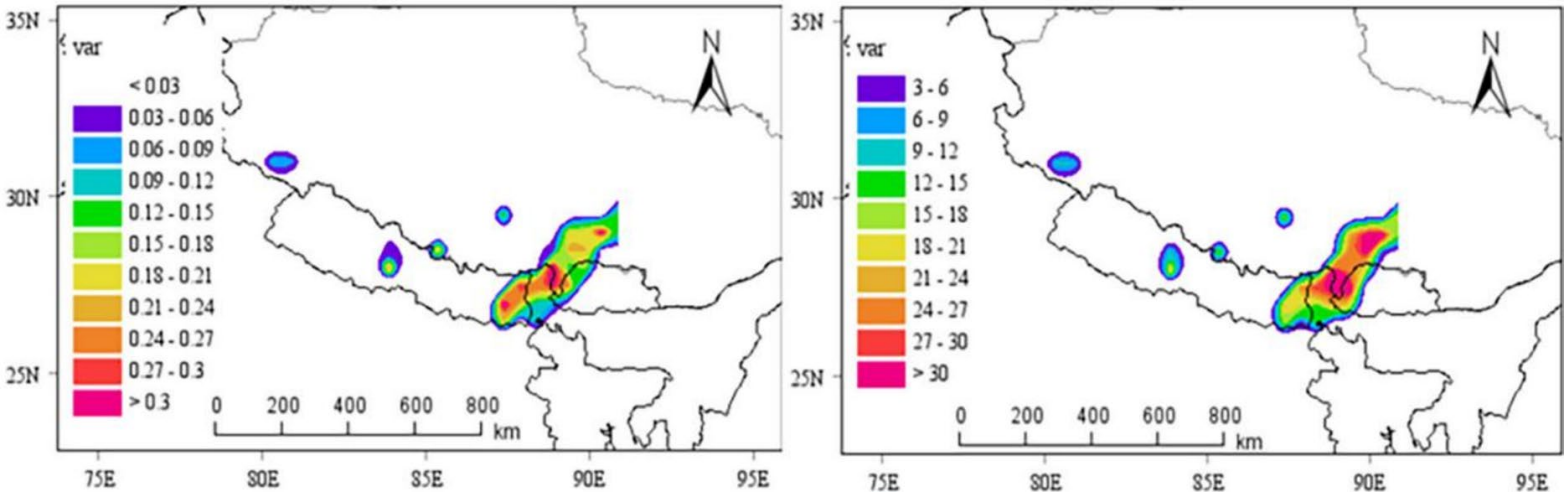
Results of PSCF and CWT analysis

To analyze the potential source area and pollution degree of atmospheric microplastics in Lhasa, PSCF and CWT were analyzed, and the simulation results were basically consistent (Fig. 7). The results showed that eastern Nepal, northeastern India, northwestern Bhutan and southeastern Tibet are all strong potential sources of pollution in Lhasa.

### Ecological risk assessment

In this study, five microplastics—PA, PS, PP, PU and PVC—accounted for up to 89.11% of the atmospheric microplastics in Lhasa. The potential ecological indices of these five microplastics in the three samples were determined, and the risk of microplastics was divided into five levels based on the RI value: a value < 150 was considered low, 100–300 was considered moderate, 300–600 was considered high, 600–1200 was considered dangerous, and ≥ 1200 was considered high risk (Table 2).

**Table 2:** Potential ecological risk assessment of the SAMPs in the study area

Sample	Hazard index	LS01	LS02	LS03
E_PA_	47	7.29	6.64	7.94
E_PS_	30	1.25	1.74	0.69
E_PP_	1	0.0009	0.004	0.002
E_PU_	7384	34.19	27.35	29.06
E_PVC_	10,001	9.26	37.04	9.26
RI		51.99	72.77	46.95
Risk level		Low	Low	Low

The assessment results showed that the E values corresponding to PVC and PU microplastics are relatively high, especially for PVC, which is very dangerous. The E values of the other types were all less than 150, indicating a low risk level. The ecological risk index of atmospheric microplastics in Lhasa ranged from 46.95 to 72.77, indicating a low risk. The LS02 risk index was the highest, as the day of sample collection was the Tibetan New Year when the atmospheric environment was affected by local activities. At present, there is no unified standard risk assessment method for microplastics and the reference values for the potential risk index method are not uniform, leading to difficulties in assessment. Therefore, there is an urgent need to establish a unified and standard ecological assessment method. In addition, the risk posed by PVC is high, so the control of PVC-type plastic products should be strengthened from the source.

## Conclusion

In this study, we have investigated the abundance, morphological characteristics, polymer composition, and potential sources of atmospheric microplastics during Tibetan New Year in Lhasa. The resulst indicated that during the days surrounding the Tibetan New Year, approximately 4.32–8.77 MPs m^−3^ were observed in Lhasa. PA and PS dominated the SAMPs, accounting for approximately 68.73% and 16.61%, respectively. The atmospheric microplastics predominantly consisted of smaller sized particles, with those less than 50 μm in diameter accounting for up to 86%. The most common shape of microplastics was fragment. And the abundance was significantly higher and more polymer components were detected on the day of the Tibetan New Year, indicating that the release of biomass and fireworks may have a certain impact on the abundance and composition of atmospheric microplastics in the region. In addition, the backwards trajectory analysis shows that the area is affected by external transportation in addition to local pollution. Therefore, long-term monitoring systems should be establised on a global scale to monitor and measure the migration and changes in atmospheric microplastics in this remote high-altitude city. The potential ecological risk index indicates that an overall risk level of microplastics in the atmospheric environment of the region is relatively low. It is important to note that our study collected fewer samples, had a limited time span, and lacked long-term monitoring of atmospheric microplastics in the region. In future work, we will conduct high-frequency sampling of MPs and expand the sampling range to collect more detailed surrounding environment data to verify the relationship between various environmental factors and MPs distribution.
